# Does Social Medical Insurance Achieve a Poverty Reduction Effect in China?

**DOI:** 10.3389/fpubh.2021.800852

**Published:** 2022-01-12

**Authors:** Ji-Le Sun, Ran Tao, Lei Wang, Li-Min Jin

**Affiliations:** ^1^School of Economics, Qingdao University, Qingdao, China; ^2^Qingdao Municipal Center for Disease Control and Prevention, Qingdao, China; ^3^School of Management, Lanzhou University, Lanzhou, China; ^4^Lanzhou Vocational and Technical College of Resources and Environment, Lanzhou, China

**Keywords:** causality, social medical insurance, poverty reduction, time-varying, subsample rolling window model

## Abstract

This paper aims to explore the impact of social medical insurance (SMI) on poverty reduction (PR) in China. Considering the time-varying characteristics of factors, this paper uses the bootstrap Granger full sample causality and subsample rolling window model to find the relationship between SMI and PR. The results highlight that in some periods, there is a bidirectional causal link between SMI and PR. Influenced by the medical insurance reform and medication measures. Social medical insurance does not have a positive impact on poverty reduction in some periods. These results are supported by the Utility Maximization Model of Insurance Consumption, which highlights that individuals make utility maximization choices when choosing insurance. The effect of medical insurance on poverty alleviation depends on whether an individual's investment in medical insurance can maximize its utility. If the proportion of social medical insurance reimbursement is too low, individuals will give up buying social medical insurance. Thus, the anti-poverty effect of social medical insurance is difficult to achieve. Therefore, authorities need to pay attention to specific contexts and social medical insurance policies and further improve the social medical insurance system to promote the realization of the anti-poverty of social medical insurance.

## Introduction

This paper aims to analyze the causality between social medical insurance (SMI) and poverty reduction (PR). The relationship between poverty and disease is complex and has been well-documented (1). On the one hand, low-income people usually lack basic health care and have poor health outcomes (2), which in turn lead to disease. On the other hand, huge medical expenses caused by diseases that are usually defined as “catastrophic” consequentially drive families into poverty (3). Disease has become one of the leading causes of household impoverishment in China (4). In 2008, 34.5% of households that were officially identified as poor by the local government, with disease declared as the main reason for their poverty. In rural areas, the situation is worse, and the proportion of poverty caused by disease rises to 37.8% (5). In 2017, the proportion of poor people who were impoverished due to major illness was ~45% (6). The World Health Organization (WHO) and other non-governmental organizations have devoted investment in health to reduce poverty (7). Since 2000, many developing countries have sought to establish universal health insurance schemes for their nations (8). By subsidizing many of the costs associated with medical care, medical insurance can provide important financial benefits to low-income families and alleviate poverty caused by disease (9). To address uncertainty in health care, China begins to establish universal medical insurance coverage (5). In 1998, urban worker basic medical insurance (UWBMI) was established, and in 2003, a new rural cooperative medical scheme (NRCMS) was established. In 2007, the benefit package of NRCMS was extended to outpatient care, and an urban residents medical insurance system (URMIS) was established. In 2016, the integration of URMIS and NRCMS was launched. The main goal of the systems above is to help rural or urban residents gain access to more health services and alleviate their financial burdens when they experience a serious illness (10). In 2013, ~95.3% of poor or subsistence allowance households were covered by social medical insurance (10). In 2020, ~98.5% of poor or subsistence allowance households are covered by social medical insurance. China has essentially achieved full medical insurance coverage for the impoverished population. However, studies demonstrate that the rapid escalation of medical expenses actually weakens the true effect of this coverage (11). The Chinese people are still burdened by high out-of-pocket (OOP) payments. Spending on private health care in China is much higher than in OECD countries. The average OOP payment of OECD countries is 10%, while it is 33.2% in China. According to an analysis of China's health service survey in 2013, the annual OOP payment rate of hospitalization expenses for the low-income population is 48.0% (9). This paper aims to determine whether social medical insurance has a positive impact on the alleviation of poverty.

Previous studies show that social medical insurance can reduce the catastrophic health expenditure risk that families face, and it has the effect of poverty reduction (12). The improvement of the medical insurance system can reduce residents' OOP expenditures, thus avoiding the poverty caused by catastrophic expenditures (13). The improvement of the medical insurance system can also improve individual health conditions, thus increasing the labor supply and reducing the probability of individuals falling into poverty (14). According to the life cycle and the persistent income hypothesis, people always save part of their income as a precautionary measure against future uncertain risks. Due to the improvement of the social medical insurance system, people reduce precautionary savings to a certain extent and increase investment in human and material capital (14). Investment in human and material capital can increase income and alleviate poverty caused by disease. Wagstaff and Lindelow (15) report that the medical expenditure of those covered by the NRCMS decreases after the introduction of the NRCMS. Zhang and Cheng (16) also find that in terms of inpatient care, the SMI helps to relieve the financial burden on the household, especially those who are in low-income groups. However, dissenters estimate that medical insurance has no significant impact on anti-poverty (17). Medical expenses continue to expand, but the reimbursement ratio of medical insurance is still relatively low. High OOP medical expenses exceed the affordability of residents; therefore, the poverty reduction effect of social medical insurance is poor (18). Yang (10) finds that insurance reimbursements do not reduce the incidence of health payment-induced poverty. As the poverty alleviation performance of basic medical insurance has heterogeneity in urban and rural areas, it has little effect on poverty reduction in deeply poor groups. Mao (17) argued that medical insurance expenditures do not provide adequate financial protection for rural households. The reimbursement ratio of NRCMS and URMIS is lower than the reimbursement ratio of UWBMI (19).

Many studies have focused on the impact of social medical insurance on poverty reduction. Many studies have concluded that health insurance has a limited effect on poverty reduction. Since there are various external events in the process of medical insurance implementation, the impact of SMI on PR may change. Previous studies ignore parameter instability caused by structural changes and may mislead the conclusions. China experiences clear economic reconstruction and structural changes. In February 2015, China began to implement centralized drug procurement in public hospitals. In July 2015, serious disease insurance was fully implemented for urban and rural residents. In January 2016, China integrated the basic medical insurance system for urban residents and the new rural cooperative medical care system and established a unified basic medical insurance system for urban and rural residents. In the same year, the remote medical settlement system was launched. In October 2018, 17 anticancer drugs were included in the drug catalog of national basic medical insurance, work-related injury insurance and maternity insurance. In December 2018, China launched a national pilot program to pay for disease diagnosis-related groups (DRGs). All of these medical insurance reforms lead to structural changes in social medical insurance and poverty reduction. Therefore, dynamic linkage between the two series would display instability. This paper takes a full account of the time-varying character that may exist in the causal link between SMI and PR. We use a bootstrap Granger full-sample causality and subsample rolling-window method to study the relationship between SMI and PR (20). This paper uses monthly data from January 2013 to December 2020. The empirical results indicate that SMI does not have a positive impact on PR in all periods. This is not consistent with previous studies. SMI precisely has a positive impact on PR when external and internal shocks are suffered. In the periods of 2015:3-2015:4 and 2018:10-2018:12, there is bidirectional negative causality between SMI and PR. This means that with the decrease in the poverty population and the increase in income, medical insurance expenditures also increase. At the same time, with the increase in medical insurance expenditure, the incidence of poverty decreases. Therefore, to achieve the anti-poverty effect of SMI, policy-makers should pay more attention to specific backgrounds (e.g., poverty reduction action and medical insurance reform) and achieve the poverty alleviation effect of social medical insurance by improving the security level of medical insurance and accelerating the integration of the medical insurance system. The empirical results also show that PR has a negative impact on SMI in some periods. This conclusion is consistent with the utility maximization model of insurance consumption, which indicates that with the increase in family income, family insurance consciousness will be gradually enhanced, and the family's ability to buy insurance will also be enhanced (15, 18–20).

The remaining sections proceed as follows: section Literature Review reviews the existing literature about the relationship between SMI and PR. Section Theoretical Model presents the Utility Maximization Model of Insurance Consumption. Section Methodology introduces the bootstrap rolling window method. Section Data and Empirical Results describes the data and empirical results. Section Conclusions offers a conclusion.

## Literature Review

Sommers and Oellerich (9) shows that medication is becoming one of the top three anti-poverty programs in the United States by reducing out-of-pocket health expenditures and reducing the poverty rate for children, adults and the elderly. Korenman and Rernler (7) indicate that the implementation of insured medical insurance significantly reduced the health poverty rate. Abay (21) demonstrates that medical insurance plays an important role in improving health care coverage and resisting families from catastrophic OOP medical costs. Wherry et al. (1) showed that the nation's public medical insurance programs have many important short- and long-term poverty-reducing benefits for low-income families with children in the United States. Fogel (22) suggested that medical insurance can improve individual health conditions, thus increasing the labor supply and reducing the probability of individuals falling into poverty. Aryeetey (23) found that in Ghana, enrollment in the National Health Insurance Scheme reduces out-of-pocket expenditures of medical expenses, provides economic protection against catastrophic expenditures, and thus reduces poverty. Bruce (24) finds that the universal medical insurance plan has a positive anti-poverty effect, but its effect on poverty reduction is not the same in different states of the United States. However, some studies show that medical insurance has no impact on poverty reduction. Kumar et al. (25) estimated that hidden poverty's rate increases with the growth of OOP health expenses in India and the anti-poverty effect of social medical insurance is poor. Wang and Xu (26) proved that the medical insurance system doesn't play as a protective role of poverty. Conversely, the medical insurance system is likely to increase the medical burden. Wagstaff and Lindelow (27) find that medical insurance significantly increases the risks of catastrophic expenditures. Shahrawat and Rao (28) showed that in some developing countries, such as Thailand, Brazil, India and South Africa, the effectiveness of social medical insurance programs in achieving equitable financing is unclear.

Zhang (29) believes that medical insurance protects against the difficulty of paying for illness of residents, and anti-poverty can be realized from the perspective of eliminating health poverty. Callander et al. (30) indicated that joining the social medical insurance plan can reduce out-of-pocket expenditures of medical expenses, provide economic protection against catastrophic expenditures, and thus reduce poverty. Yang et al. (31) showed that the effect of health payments on poverty was more severe, while the financial protection effect of the NRCMS was stronger. NRCMS could achieve the policy target of alleviating health payment-induced poverty. Zhai et al. (32) found that NRCMS has a positive effect on alleviating poverty among rural older adults. Ma et al. (33) showed that medical insurance schemes are insufficient for protection against the economic burden of middle-aged and elderly people in China. Jing et al. (34) suggested that NRCMS substantially reduces the financial burden for older adults with chronic diseases. Zhou et al. (35) showed that NRCMS can reduce poverty by significantly improving the agricultural labor hours of the insured. Mateusz et al. (36) found that low-income families benefit more from medical insurance. However, some studies indicate that medical insurance plays a small role in poverty reduction in China. Dai (37) argues that the impact of social medical insurance is very limited for families suffering catastrophic expenditures due to chronic diseases. Sun et al. (38) showed that at lower levels of health services hierarchy, doctors tend to overprescribe for patients covered by NRCMS, which leads to the inflation of medical costs. Yang (10) found that the implementation of NRCMS does not reduce poverty but makes low-income people fall into the poverty trap. Bai and Wu (39) indicated that there is significant heterogeneity in the direction and magnitude of the impact of NRCMS on the income of different subgroups. The income level of the lowest income group and a few highest groups is damaged after participation, while middle- and high-income groups benefit from participation. Shi et al. (40) studied the weak effect of public transfer income, such as medical insurance, on poverty reduction.

Previous studies on the anti-poverty reduction of social medical insurance have not considered the external impact of China's economic structure transformation and medical insurance system reform. Thus, the conclusions are unreliable. The Granger causal relationship between SMI and PR is time-varying if structural changes exist. To ensure the reliability of the research conclusions, this paper uses the bootstrap subsample rolling Windows Granger causality test to find the relationship between SMI and PR.

## Theoretical Model

Shi et al. (40) indicated that the role of social medical insurance in poverty alleviation largely depends on the proportion of medical assistance and OOP. If the proportion of OOP is too high, individuals may choose not to buy health insurance or choose commercial insurance. Individuals make utility maximization choices when choosing insurance. The effect of medical insurance on poverty alleviation depends on whether an individual's investment in medical insurance can maximize its utility. Benjamin (7) presents a utility maximization model of insurance consumption about the insurance type choice of individuals who are eligible for medication. Individuals' choice of insurance type is based on the distribution of their possible OOP expenditures. The model sets three distinct insurance consumption states: covered by Medicaid, choice of business medical insurance, and without any insurance in the framework of expected utility. Benjamin (7) treats the individual's utility as a function of non-medical insurance consumption and an effective premium. At the same time, Benjamin adds two variables, income and medical OOP spending, to the model.

We replace Medicaid coverage with medical insurance, which includes UWBMI, URMIS and NRCMS. We further adopt the model, given the equation:


(1) $$\begin{array}{l} Max\int u\left(Y-S-P_{T}\right)f_{T}\left(S\right)dS \end{array}$$

where an individual's insurance consumption utility u(C, P) is a function of non-medical insurance consumption C and an effective premium P. Y represents income before offering premiums and paying any OOP medical. S is a non-negative random variable and represents an individual's medical OOP spending. The function f(S) is the probability density function of S. The function f(–) depends on the type of insurance consumption, T: medical insurance, choice of business medical insurance, or without any insurance. When all consumers are assumed to be risk adverse, u(–) is concave. The individual can maximize his expected utility of insurance consumption by choosing insurance type T. As seen from Equation (1), a high proportion of OOP will directly affect the utility of individuals. To maximize utility, individuals may give up purchasing medical insurance, so the poverty alleviation effect of medical insurance will be difficult to achieve.

**Figure 1:**
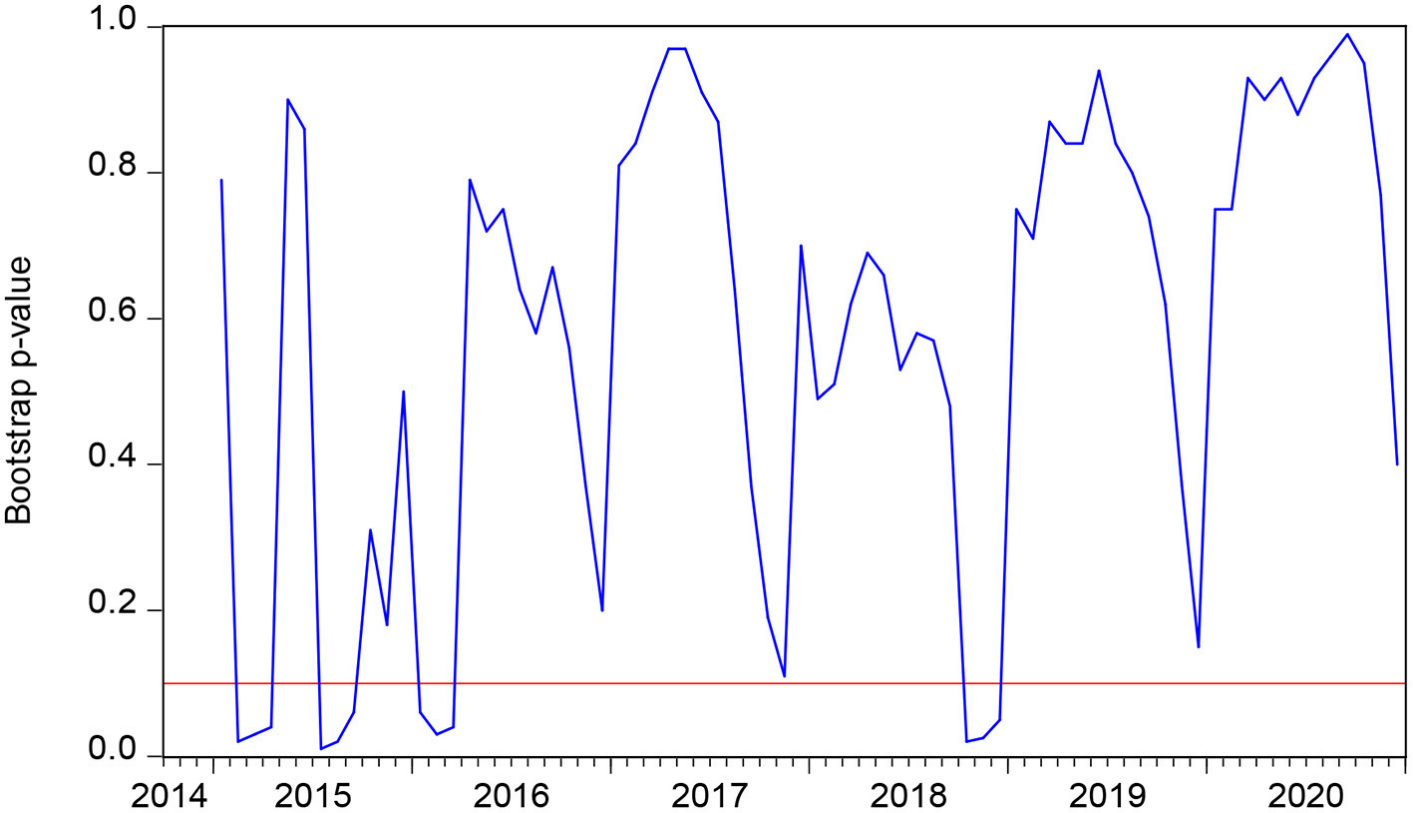
The bootstrap *p*-value of the rolling test statistic testing the null that SMI does not Granger cause PR.


Figure 1 describes the link among factors.

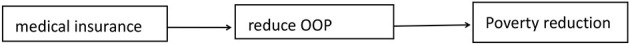


Summarizing the above analyses, we put forward the following Hypotheses I and II.

Hypothesis I: If medical insurance can reduce the proportion of individual out-of-pocket expenses, so that individuals can achieve the maximum utility, and then achieve the effect of poverty reduction.
Hypothesis II: If medical insurance cannot reduce the proportion of individual out-of-pocket expenses and maximize individual utility, individuals will give up buying medical insurance or choose commercial insurance. Medical insurance does not reduce poverty.

## Methodology

### Bootstrap Full-Sample Causality Test

Based on the traditional vector autoregression (VAR) model, the Granger causality test statistics cannot obey the standard asymptotic distributions. To improve the Granger causality test and prevent inaccurate results, Shukur and Mantalos (41) develop the residual-based bootstrap (*RB*) method. They highlight that the *RB* method is more reliable for causality tests with standard asymptotic distributions. In addition, the *RB* method can be applied in small samples. Shukur and Mantalos (42) also developed likelihood ratio (*LR*) tests that can be modified by the characteristics of power and size. The present study employs the *RB*-based modified *LR* statistic to examine the causality between SMI and PR. A vector autoregressive (VAR) model is as follows:


(2) $$\begin{array}{l} Y_{t}=\alpha_{0}+\alpha_{1}Y_{t-1}+......+\alpha_{p}Y_{t-p}+\upsilon_{t}\\ \;\text{t}=1,2,\ldots ,\text{T} \end{array}$$

where we choose the optimal lag order p based on the Schwarz information criterion (SIC). *Y* can be split into SMI and PR in a two-variable VAR (*p*) process, that is, $Y_{t}={\left(\text{SM}{\text{I}}_{t},\text{P}{\text{R}}_{t}\right)}^{\prime }$. According to the Utility Maximization Model of Insurance Consumption (7), to maximize insurance utility, individuals will reduce their purchase of social medical insurance when social assistance increases. There is a substitution relationship between social assistance and social medical insurance. In addition, social assistance has a positive impact on poverty reduction. Social assistance can directly increase the disposable income of low-income groups, thereby reducing poverty. Hence, we choose social assistance (SA) as a control variable. Equation (2) can be rewritten as follows:


(3) $$\begin{array}{l} \left[\begin{array}{c} \text{SM}{\text{I}}_{\text{t}}\\ \text{P}{\text{R}}_{\text{t}} \end{array}\right]=\left[\begin{array}{c} \alpha_{10}\\ \alpha_{20} \end{array}\right]+\left[\begin{array}{ccc} \alpha_{11}\left(O\right) & \alpha_{12}\left(O\right) & \alpha_{13}\left(O\right)\\ \alpha_{21}\left(O\right) & \alpha_{22}\left(O\right) & \alpha_{23}\left(O\right) \end{array}\right]\left[\begin{array}{c} \text{SM}{\text{I}}_{\text{t}}\\ \text{P}{\text{R}}_{\text{t}}\\ \text{S}{\text{A}}_{\text{t}} \end{array}\right]\\ +\left[\begin{array}{c} \upsilon_{1t}\\ \upsilon_{2t} \end{array}\right] \end{array}$$

where $\upsilon_{t}={\left(\upsilon_{1t},\upsilon_{2t}\right)}^{\prime }$ is a white-noise process with zero mean and covariance matrix. The lag operator O can be expressed as $O^{k}Y_{t}=Y_{t-k}$, $\alpha_{ij}\left(O\right)=\sum_{k=1}^{p}\alpha_{ij,k}O^{k}$, *i* = 1, 2, *j* = 1, 2, 3.

According to Equation (3), the null hypothesis that SMI does not Granger cause PR is α_12, *k*_ = 0 where *k* = 1, 2…, p. If SMI can affect PR, this null hypothesis can be rejected and vice versa. The inverse null hypothesis that PR does not Granger cause SMI is α_21, *k*_ = 0, where *k* = 1, 2…, p. The null hypothesis can be rejected if PR can affect SMI and vice versa.

### Parameter Stability Test

The VAR model parameter will show instability when structural changes occurred in full-sample data. Hence, the full-sample test may lead to incorrect conclusions if the parameters are unstable. To test the parameter stability, Andrews (43) and Andrews and Ploberger (44) developed the *Sup-F, Ave-F* and *Exp-F* tests. The *Sup-F* test was developed to recognize the sudden structural changes. *Ave-F* and *Exp-F* tests are developed to recognize whether the parameters have a tendency to evolve over time. To test whether the parameters follow a random walk process, this paper uses the *Lc* statistics test (44, 45). All of the abovementioned tests can prove the parameter stability. If the parameter has demonstrated instability, the Granger causality between SMI and PR is time-varying. Therefore, the subsample test is applied to research the Granger causality between SMI and PR in this paper.

### Subsample Rolling Window Test

The subsample rolling window test, which was developed by Balcilar et al. (46), can divide the whole time series into small samples on the basis of the rolling window width. However, it is not easy to choose the rolling window width. A small value can increase the representativeness and reduce the standard error of estimations but may reduce the accuracy of estimation, while a large value may increase the accuracy of estimations but may reduce the representativeness and increase the standard error of estimations. To achieve the trade-off between exactitude and typicality, Pesaran and Timmermann (47) point out that the width of the rolling window should not be <20. Suppose the length of the time series is *T* and the rolling window width is *w*. The end of each divided small sample is *w, w*+*1…, T*. We finally obtain *T-w*+*1* subsamples. Then, we use *RB*-based modified *LR* tests to identify the subsample causality. The bootstrap subsample rolling-window Granger causality test result can be obtained from all the *p*-values of *LR* statistics rolled by *T-w* +*1* subsamples. The average of a large number of estimations $N_{b}^{-1}\sum_{k=1}^{p}{\hat{\alpha }}_{12,k}^{*}$ (*N*_*b*_ is the frequency of the repetitions, ${\hat{\alpha }}_{12,k}^{*}$ is bootstrap estimates in the VAR system) reveals the impact of PR on SMI. The average of a large number of estimations $N_{b}^{-1}\sum_{k=1}^{p}{\hat{\alpha }}_{21,k}^{*}$ (*N*_*b*_ is the frequency of the repetitions, and ${\hat{\alpha }}_{21,k}^{*}$ is bootstrap estimates in the VAR system) reveals the effect from SMI to PR. Meanwhile, according to Balcilar (46), the lower and upper bounds of a 90% confidence interval are calculated by the 5th and 95th quantiles of ${\hat{\alpha }}_{12,k}^{*}$ and ${\hat{\alpha }}_{21,k}^{*}$, respectively.

## Data and Empirical Results

This paper uses monthly data from January 2013 to December 2020 to analyze the Granger causal relationship between social medical insurance and poverty reduction in China. In 2013, China entered the transition period of economic structure. Poverty caused by unemployment and disease increased. Since the 18th National Congress of the Communist Party of China (CPC), China has made decisive progress in poverty alleviation by taking targeted measures to alleviate poverty. In 2013, the Ministry of Finance and the Ministry of Civil Affairs formulated the Administrative Measures for Urban and Rural Medical Assistance Funds. The government intends to reduce poverty by increasing medical assistance based on existing medical insurance. Hence, we choose the premium income of social medical insurance as the proxy index of social medical insurance (38, 39, 42) and we choose number of persons covered by the subsistence allowances as the proxy index of poverty reduction (38, 41, 43). The data sources are from the Wind database.

To test the stability of SMI, PR and SA, our study employs the augmented Dickey-Fuller (48) test, Phillips-Perron (49) test and Kwiatkowski-Phillips-Schmidt-Shin (50) test. Table 1 describes the results in which all three variables are I (1). Therefore, this paper constructs VAR models with the first differences of SMI, PR and SA to ensure the stationarity of the data and the reliability of Granger causality tests.

**Table 1:** Unit root tests.

	**ADF**	**PP**	**KPSS**
SMI	2.355 (11)	−2.785 (2)	4.077^***^ (8)
PR	0.408 (1)	0.768 (1)	4.328^***^ (1)
SA	1.067 (3)	0.796 (3)	3.564^***^ (2)
ΔSMI	−4.513^***^ (11)	−10.350^***^ (1)	1.058 (1)
ΔPR	−4.571^***^ (0)	−4.590^***^ (1)	1.126 (1)
ΔSA	−5.675^***^ (1)	−6.875^***^ (2)	1.089 (1)

*** *Indicate significance at 1% levels. The numbers in parentheses indicate the lag order selected based on the recursive t-statistic, as suggested by Perron (51)*.

Equation (3) shows the bivariate VAR model this paper constructs. Based on the Schwarz information criterion (SIC), we choose the optimum lag length of SMI and PR at a value of 7. According to the *RB*-based modified *LR* tests, the full-sample causality results are indicated in Table 2. The bootstrap *p*-value indicates that the null hypothesis of the causality test is rejected, which means that poverty reduction does Granger cause social medical insurance. This finding is in line with studies (38, 50). They indicate that low levels of poverty and high relative incomes lead to increased spending on medical insurance. However, the null hypothesis of the causality test that SMI does not Granger cause PR is not rejected, suggesting that social medical insurance does not Granger cause poverty reduction. This conclusion is in line with studies (52–54). It can be supported by the Utility Maximization Model of Insurance Consumption (7), which states that if the proportion of OOP is too high, individuals may consider giving up buying insurance for utility maximization. Thus, social medical insurance cannot reduce poverty.

**Table 2:** Full-sample Granger causality tests.

	**H0:SMI does not Granger cause PR**	**H0:PR does not Granger cause SMI**
**Bootstrap LR**		
Test	6.032	25.617^***^

*We calculate p values using 10,000 bootstrap repetitions.*

*** *Indicate significance at 1% levels*.

However, previous studies on the causal relationship between SMI and PR assume that there are no structural changes in time series and that the causal relationship between variables is constant over the entire sample period. In the presence of structural changes, the parameters estimated utilizing SMI and PR will change. Therefore, the Granger causality between SMI and PR may be volatile with time. The conclusions of the full-sample tests are no longer reliable (9). Therefore, we conduct the *Sup-F, Mean-F* and *Exp-F* tests to examine the stability of the parameters and identify if there are structural changes (55). Table 3 shows all results. In the first row, the results derived from the *Sup-F* test show that there is a one-time sharp shift appearing in the SMI, PR and VAR systems at the 1 percent level. In the second row, the results derived from the *Mean-F* test show that equations from the SMI, PR and VAR systems may gradually change over time. In the third row, the results from the *Exp-F* test have the same conclusion as the *Mean-F* test. We synchronously conduct the *Lc* test to examine all parameters that appeared in the VAR system. The results from the *Lc* test testify that the parameters in the overall VAR models follow the random walk process and are non-constant. Therefore, due to structural changes, parameters show short-term instability.

**Table 3:** Parameter stability tests.

	**SMI equation**	**PR equation**	**VAR system**
Sup-F	49.912^***^	80.743^***^	48.903^***^
Mean-F	27.089^***^	28.086^***^	23.803^***^
Exp-F	9.785^***^	36.280^***^	20.393^***^
LC			9.556^***^

*We calculate p values using 10,000 bootstrap repetitions*.

*** *Denote significance at 1%*.

*Hansen-Nyblom (Lc) parameter stability test for all parameters in the VAR jointly*.

Considering the structural changes that exist, the VAR model estimated is volatile in the full sample. The rolling-window technique, which considers the structural changes and allows the causal links between the two variables SMI and PR to be time-varying across various subsamples, is different from the VAR model. Therefore, this paper employs the rolling-window technique to find the relationship between SMI and PR. We can estimate the bootstrap *p*-values of *LR* statistics from the VAR models in Equation (3). These rolling estimates move from 2015:01 to 2020:12 after trimming 24-month observations^1^ from the beginning of the null sample.

**Figure 2:**
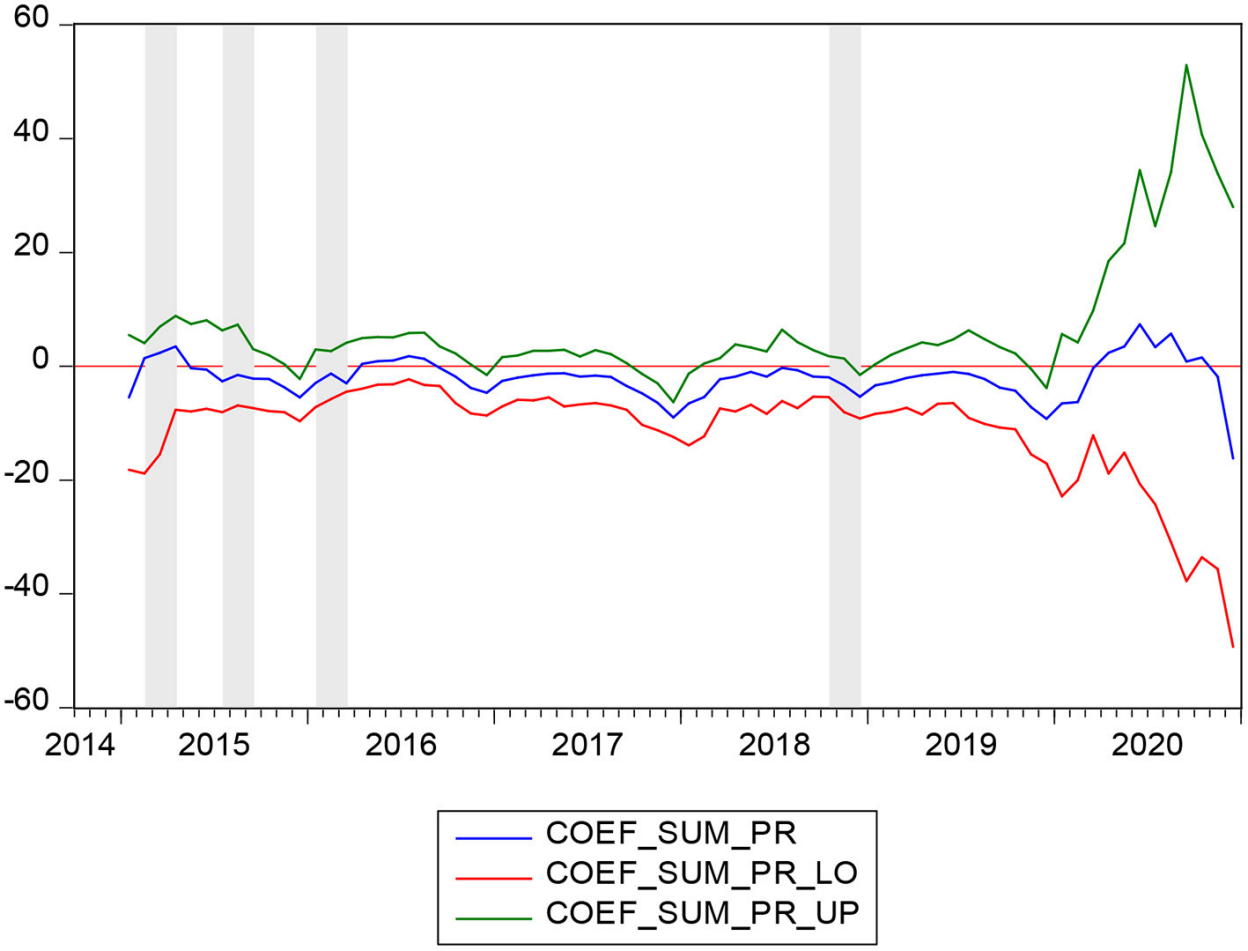
Bootstrap estimates of the sum of the rolling-window coefficients for the impact of SMI on PR.

Figure 1 highlights the rolling bootstrap p values. As the null hypothesis that SMI does not Granger cause PR at 10 percent is rejected, we can easily conclude that SMI can interpret the alterations in PR to some extent across the subperiod. Figure 2 shows the bootstrap estimations based on the summation of rolling window coefficients with which we can easily obtain the effect of SMI on PR. As we can obtain from Figure 1 that in the periods 2015:02-2015:04, 2015:07-2015:09, 2016:01-2016:03, and 2018:10-2018:12 the null hypothesis can be rejected. Figure 2 shows that in 2015:02-2015:04, SMI has a positive impact on PR. In 2015:07-2015:09, 2016:01-2016:03, and 2018:10-2018:12, the SMI had a negative impact on PR.

For a long time, the drug procurement of public hospitals lacks unified management, and most of the procurement is conducted by hospitals as individuals. Due to the lack of supervision and driven by interests, pharmaceutical production and management enterprises bribe the management staff of major hospitals and inflate drug prices. This further places a financial burden on low-income groups and drives them into poverty. In February 2015, the General Office of the State Council issued the Guideline on Improving Centralized Drug Procurement in Public Hospitals. The guideline stipulates that all drugs used in public hospitals (excluding TCM decoction pieces) should be purchased through provincial centralized drug procurement platforms. This measure accelerates the reform of public hospitals and reduces inflated drug prices and the burden of drugs on residents (56). However, before the formal implementation of the system, the original beneficiaries, including drug sellers and hospital purchasers, are bound to take advantage of the last opportunity to increase the export of illegal benefits. Hence, in 2015:02-2015:04, the SMI had a positive impact on PR. The increase in social medical insurance premiums is accompanied by an increase in poverty (57).

The huge medical expenses caused by serious diseases are an important cause of poverty (58). In July 2015, the General Office of the State Council issued opinions on the full implementation of serious illness insurance for urban and rural residents. It states that by the end of 2015, serious disease insurance will cover all persons participating in urban residents' basic medical insurance and the new rural cooperative medical care system. The full implementation of serious illness insurance effectively reduces the medical burden of patients with serious diseases. An increase in the payment proportion of serious illness insurance can reduce the burden of individual medical expenses more effectively (31). Hence, this measure directly reduces the poverty. This can be supported by the Utility Maximization Model of Insurance Consumption (7), which states that if the proportion of OOP is not too high, individuals may consider buying insurance for utility maximization. Because of the new rules on serious illness insurance, during the period of 2015:07-2015:09, the SMI had a negative impact on PR. The increase in social medical insurance premiums is accompanied by a decrease in poverty.

Before 2016, China implemented a cooperative medical system in rural areas that was different from the urban medical insurance system. However, due to many restrictions on farmers' income and finance at the township level, the problem of high OOP for farmers is not well-solved, and the effect of poverty reduction relying on medical insurance is not achieved (59). In January 2016, the State Council put forward clear requirements on the integration of the basic medical insurance system for urban residents and the new rural cooperative medical system. The establishment of a unified basic medical insurance system for urban and rural residents advances the reform of medical and health care systems. This ensures that urban and rural residents can enjoy equal rights and interests in basic medical insurance. After the implementation of unified urban and rural medical insurance, rural people can enjoy the same medical security treatment as urban residents, including the minimum payment standard, reimbursement ratio and maximum payment limit. This is of great significance for reducing farmers' self-payment ratio and alleviating poverty. During the period of 2016:01-2016:03, the SMI had a negative impact on PR. The increase in social medical insurance premiums is accompanied by a decrease in poverty.

In October 2018, the State Medical Insurance Administration issued a notice on the inclusion of 17 anticancer drugs in the National Basic Medical Insurance, Industrial Injury Insurance and Maternity Insurance drug Catalog categories. These measures further reduce the drug burden for cancer patients and families. In December 2018, the State Medical Insurance Administration issued a notice on the National Pilot Program of Payment by Diagnosis-related Groups (DRGs), proposing to accelerate the national pilot program of payment by DRGs and explore the establishment of a DRG payment system. DRGs link the hospital's treatment of patients with the cost incurred, effectively improving the comparability between different providers of medical services. It provides a unified measure of medical service output between various specialties within the hospital and physicians within the same specialty. At the same time, it also provides a basis for the formulation of payment standards, especially the implementation of prepayment, to facilitate the overall evaluation and unified management of medical services. As a result of the above two reform measures, during the period of 2018:10-2018:12, the SMI had a negative impact on PR. The increase in social medical insurance premiums is accompanied by a decrease in poverty.

**Figure 3:**
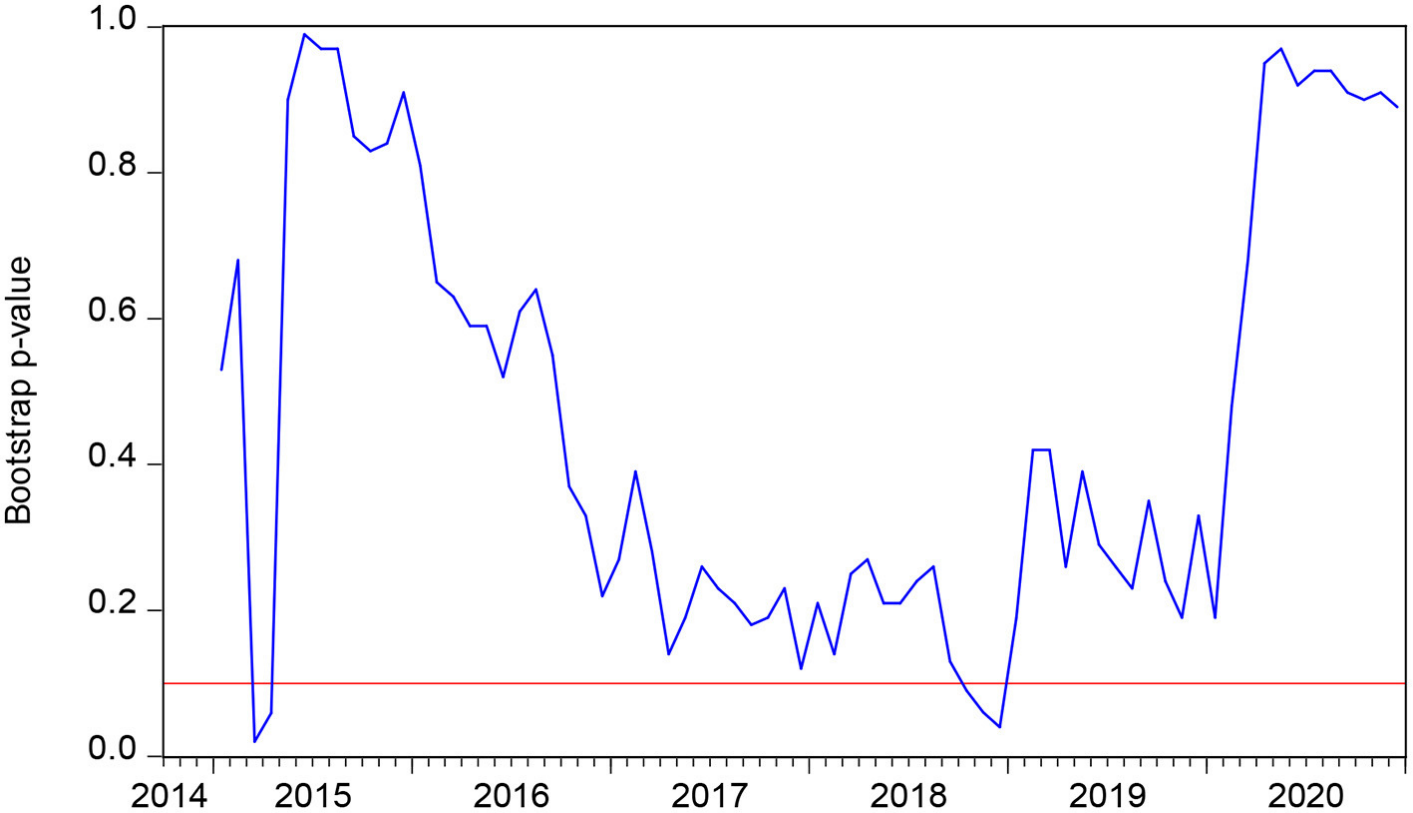
The bootstrap *p*-value of the rolling test statistic testing the null that PR does not Granger cause SMI.

**Figure 4:**
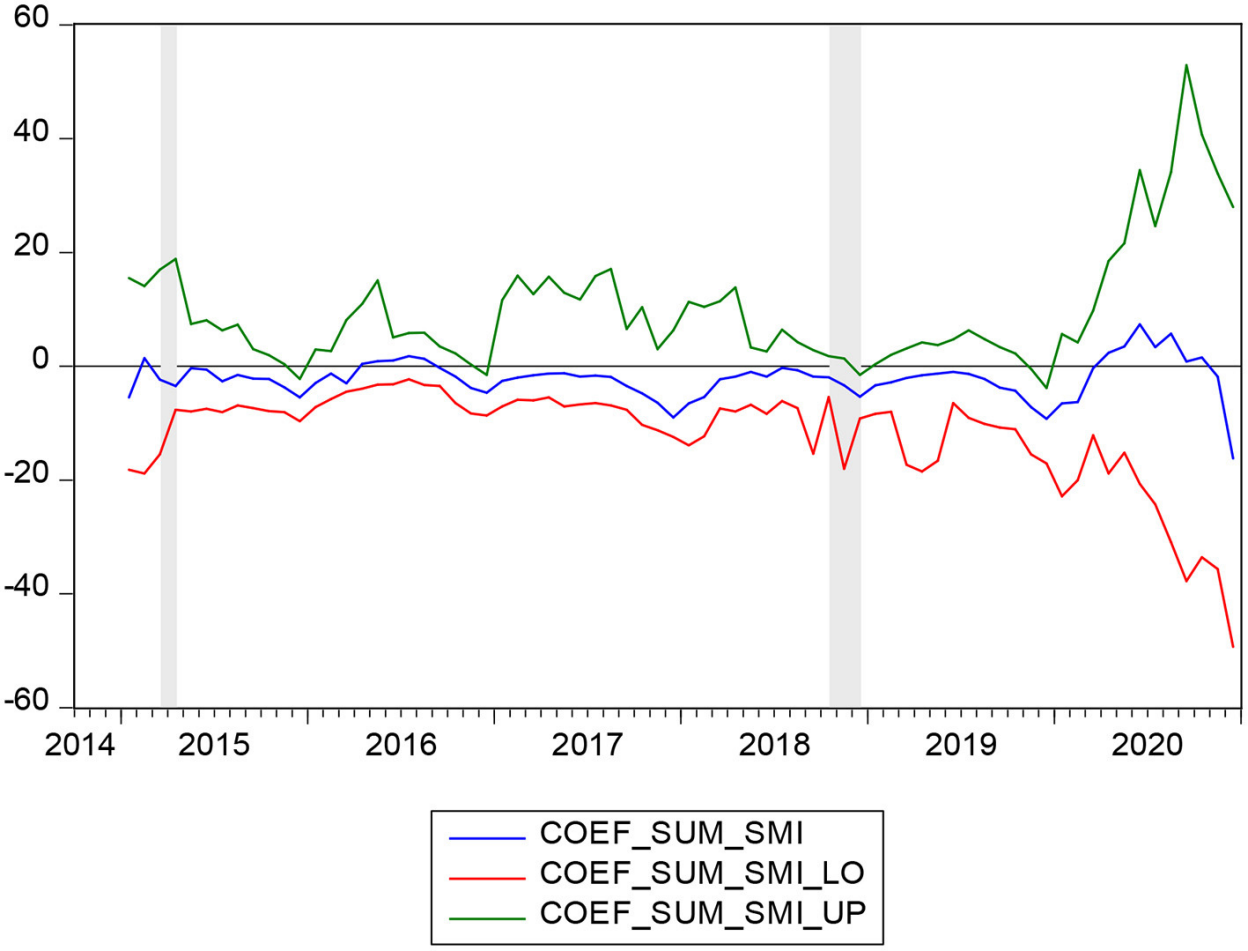
Bootstrap estimates of the sum of the rolling-window coefficients for the impact of PR on SMI.

Figure 3 highlights the rolling bootstrap *p*-values. This indicates that the null hypothesis that PR does not Granger cause SMI is rejected at 10 percent. Figure 4 shows the direction of the causality between PR and SMI, with which we can easily obtain the effect of PR on SMI. As we can obtain from Figure 3 in the periods 2015:3-2015:5 and 2018:10-2018:12, the null hypothesis can be rejected. Figure 4 indicates that in 2015:3-2015:5 and 2018:10-2018:12, PR has a negative impact on SMI. As introduced earlier, the PR is represented by the number of persons covered by the subsistence allowances. It is believed that with the increase of family income, family insurance consciousness will be gradually enhanced, and the family's ability to buy insurance will also be enhanced (8, 60). As a family's income increases, it spends a higher proportion on health insurance. However, in other periods except 2015:3-2015:5 and 2018:10-2018:12, PR does not have a significant impact on SMI, possibly because there are other factors influencing SMI instead of PR.

In February 2015, The General Office of the State Council issued the Guideline on Improving Centralized Drug Procurement in Public Hospitals. The guideline stipulates that all drugs used in public hospitals (excluding TCM decoction pieces) should be purchased through provincial centralized drug procurement platforms. This measure reduces inflated drug prices and the burden of drug use on residents. This makes residents give priority to basic medical insurance when they choose to maximize insurance consumption (7). During the period of 2015:3-2015:5, PR had a negative impact on the SMI. The decrease in poverty reduction is accompanied by an increase in social medical insurance.

Medical assistance plays an important role in poverty reduction (9, 61). In October 2018, the National Medical Insurance Administration, the Ministry of Finance and the Poverty Alleviation Office of the State Council jointly formulated the Three-year Action Plan for Poverty Alleviation through Medical Security (2018-2020). The Medicaid plan increases input in urban and rural medical assistance. For three consecutive years since 2018, the central government has allocated subsidies through medical assistance funds to improve medical security for the poor rural population in poverty-stricken areas and strengthen medical assistance to meet basic needs. The acquisition of medical assistance directly increases the disposable income of low-income groups and thus makes them have extra funds to purchase insurance. Hence, during the period of 2018:10-2018:12, PR had a negative impact on SMI. The decrease in poverty reduction is accompanied by an increase in social medical insurance.

In this paper, we adopt the bootstrap Granger full-sample causality test and subsample rolling-window estimations to find the causality between SMI and PR. We finally obtain an additional perspective on the dynamic causality between SMI and PR in China. In summary, the relationship between SMI and PR is not always consistent with the previous views that SMI has a positive impact on PR. The explanation may be that there are other factors influencing SMI instead of PR. Kumar et al. (25) estimate that hidden poverty rates increase with the growth of OOP health expenses and that the anti-poverty effect of social medical insurance is poor. Sun et al. (38) shows that at lower levels of health services hierarchy, doctors tend to overprescribe for patients covered by NRCMS, which leads to the inflation of medical costs. In the periods of 2015:3-2015:4 and 2018:10-2018:12, there is bidirectional negative causality between SMI and PR. This means that with the decrease in the poverty population and the increase in income, medical insurance expenditures also increase. At the same time, with the increase in medical insurance expenditure, the incidence of poverty decreases. This means that social medical insurance does have the effect of poverty reduction.

## Conclusions

This paper explores the correlation between SMI and PR by using a bootstrap full-sample and subsample rolling-window causality estimation in China. Through bootstrap full-sample causality tests, we can conclude that there is unidirectional causality between the SMI and PR. However, taking into consideration that structural changes may exist, this paper employs a subsample rolling window causality estimation.

The results estimate that SMI does not have a positive impact on PR in some periods. These results are supported by the Utility Maximization Model of Insurance Consumption (7), which highlights that individuals make utility maximization choices when choosing insurance. The effect of medical insurance on poverty alleviation depends on whether an individual's investment in medical insurance can maximize its utility. If the proportion of social medical insurance reimbursement is too low or medical costs are inflated by overprescribing, individuals will give up buying social medical insurance (30, 41, 62). Thus, the anti-poverty effect of social medical insurance is difficult to achieve. However, in some periods, SMI does have a positive impact on PR. In July 2015, the General Office of the State Council issued opinions on the full implementation of serious illness insurance for urban and rural residents. The increase in the payment proportion of serious illness insurance can reduce the burden of individual medical expenses more effectively. In January 2016, the State Council began to establish a unified basic medical insurance system for urban and rural residents. This ensures that urban and rural residents can enjoy equal rights and interests in basic medical insurance. In October 2018, the State Medical Insurance Administration issued a notice on the inclusion of 17 anticancer drugs in the National Basic Medical Insurance, Industrial Injury Insurance and Maternity Insurance drug Catalog categories. In December 2018, the State Medical Insurance Administration issued a notice on the National Pilot Program of Payment by Diagnosis-related Groups (DRGs). The above four medical insurance reform measures significantly reduced the proportion of individual medical expenses and further improved the level of medical insurance services. Additionally, the fairness of medical insurance has been further realized. These measures are all factors that lead to the significant effect of SMI on PR. In October 2018, the National Medical Insurance Administration, the Ministry of Finance and the Poverty Alleviation Office of the State Council jointly formulated the Three-year Action Plan for Poverty Alleviation through Medical Security (2018-2020). The Medicaid plan increases input in urban and rural medical assistance. In this period, PR had a negative impact on SMI. Medical insurance expenditures increase with a decrease in the poverty population. This result is consistent with the utility maximization model of insurance consumption and the life-cycle hypothesis.

The results bring into correspondence the fact that China has experienced poverty alleviation and social medical insurance reform. To achieve a poverty reduction effect, China needs to continuously improve the security level of social medical insurance by adopting specific measures, including improving the reimbursement ratio of medical insurance, further promoting the integration of the medical insurance system, and reforming medical expenses. The medical level of urban and rural residents also has a significant impact on residents' medical tendency. China needs to further improve the level of community-level medical services and intensify efforts to train medical staff to realize the equalization of medical and health services in different regions.

The correlation between social medical insurance and poverty reduction could be a fruitful area for future study. We intend to examine the relationship between the level of economic development in different regions of China and the anti-poverty effect of medical insurance in future studies; thus, a new method (e.g., panel threshold regression model) will be utilized.

## Data Availability Statement

The original contributions presented in the study are included in the article/supplementary material, further inquiries can be directed to the corresponding author/s.

## Author Contributions

J-LS was responsible for the overall design and writing of the article. RT was responsible for collecting data. LW was responsible for sorting out data and establishing models. L-MJ was responsible for typesetting and editing. All authors contributed to the article and approved the submitted version.

## Conflict of Interest

The authors declare that the research was conducted in the absence of any commercial or financial relationships that could be construed as a potential conflict of interest.

## Publisher's Note

All claims expressed in this article are solely those of the authors and do not necessarily represent those of their affiliated organizations, or those of the publisher, the editors and the reviewers. Any product that may be evaluated in this article, or claim that may be made by its manufacturer, is not guaranteed or endorsed by the publisher.

## Footnotes

^1^To prove the reliability of the test results, this paper also uses the rolling-window widths of 20-, 28- and 32- months to explore the causality, and the results are consistent with the 24-months rolling-window.
